# Minimal residual disease in cervical cancer: biological persistence, ctHPV DNA-guided surveillance, and recurrence-directed intervention

**DOI:** 10.3389/fonc.2026.1938965

**Published:** 2026-09-22

**Authors:** Jing Wu, Teng Wang, Chunyue Sheng, Wenhui Wang

**Affiliations:** 1 Department of Obstetrics and Gynecology, Ninghai Maternal and Child Health Hospital, Ningbo, China; 2 Department of Traditional Chinese Medicine, Maternity & Child Care Center Of DeZhou, Dezhou, China; 3 Department of Thoracic Surgery, Ninghai First Hospital, Ningbo, China

**Keywords:** adoptive cell therapy, cervical cancer, circulating tumor HPV DNA, immune checkpoint inhibition, liquid biopsy, minimal residual disease, post-treatment surveillance, therapeutic HPV vaccine

## Abstract

Cervical cancer remains a leading cause of cancer-related mortality among women worldwide, with approximately 30–40% of patients experiencing recurrence after definitive chemoradiotherapy for locally advanced disease. The concept of minimal residual disease (MRD)—the persistence of viable tumor clones after curative-intent treatment that escape conventional radiographic and clinical detection—has transformed surveillance and treatment paradigms in hematological malignancies and, increasingly, in solid tumors. In cervical cancer, the near-universal association with high-risk human papillomavirus (HPV) provides a unique molecular handle for MRD detection: circulating tumor HPV DNA (ctHPV DNA) released into the bloodstream by residual tumor cells. Here, we review three interconnected dimensions of MRD in cervical cancer. First, we examine the biological basis of tumor persistence after chemoradiotherapy, encompassing HPV integration-driven genomic instability, immune evasion within the irradiated tumor microenvironment, and clonal repopulation kinetics. Second, we synthesize the rapidly maturing evidence for ctHPV DNA-guided post-treatment surveillance, from digital PCR and next-generation sequencing-based detection platforms to prospective clinical validation studies demonstrating that detectable ctHPV DNA after chemoradiotherapy independently predicts recurrence with lead times of 3–6 months before radiographic progression. Third, we evaluate emerging recurrence-directed intervention strategies, including MRD-stratified treatment intensification (immune checkpoint inhibition and therapeutic HPV vaccination), adoptive cell therapy, and how integrating ctHPV DNA dynamics with immunotherapeutic approaches may define a new framework for personalized post-treatment management in this molecularly tractable malignancy.

## 1 Introduction

Cervical cancer is the fourth most common malignancy in women globally, with approximately 660,000 new cases and 350,000 deaths annually (1). Although prophylactic HPV vaccination and organized screening programs have substantially reduced incidence in high-income countries, the burden remains disproportionately high in low- and middle-income settings, where most patients present with locally advanced or metastatic disease (1, 2). For patients with locally advanced cervical cancer (International Federation of Gynecology and Obstetrics (FIGO) stages IB2–IVA), definitive cisplatin-based chemoradiotherapy (CRT) with brachytherapy remains the standard of care, achieving 5-year overall survival rates of approximately 65–75% (3, 4). However, recurrence rates remain stubbornly elevated at 30–40%, with the majority of relapses occurring within the first two to three years after treatment (3, 5).

The central clinical challenge in post-treatment cervical cancer management is the inability of current surveillance modalities—physical examination, cytology, and cross-sectional imaging—to distinguish patients who are cured from those who harbor residual disease that will eventually manifest as clinical recurrence. Post-treatment imaging with fluorodeoxyglucose positron emission tomography/computed tomography (FDG-PET/CT) at 3 months after CRT demonstrates a complete metabolic response in the majority of patients, yet a substantial fraction of these radiographically “disease-free” patients subsequently relapse (6). This diagnostic gap reflects the presence of minimal residual disease: viable tumor cells that persist below the detection threshold of conventional modalities and are capable of driving late recurrence through clonal repopulation.

The near-universal etiological association between cervical cancer and high-risk HPV infection—with HPV16 and HPV18 accounting for approximately 70% of cases—creates a distinctive opportunity for liquid biopsy-based MRD surveillance (7–9). Unlike most solid tumors, in which MRD detection requires tracking patient-specific somatic mutations through tumor-informed sequencing panels, HPV-driven cervical cancers harbor a universally expressed viral oncogenic driver whose DNA can be detected in the circulation. The viral E6 and E7 oncoproteins are obligate drivers of the malignant phenotype; their continued expression is required for tumor cell survival, and the integrated or episomal viral DNA that encodes them is released into the bloodstream upon tumor cell death or turnover (10, 11). Detection of circulating tumor HPV DNA (ctHPV DNA) thus provides a direct, tumor-specific molecular marker of residual disease burden that is both biologically rational and technically tractable.

Over the past five years, a convergence of technological advances—ultrasensitive digital PCR assays with limits of detection below 0.1 copies per milliliter, HPV-agnostic next-generation sequencing panels, and standardized plasma collection protocols—has propelled ctHPV DNA from a promising research biomarker to a clinically validated tool for post-treatment surveillance (12–16). Prospective studies have now demonstrated that persistent ctHPV DNA positivity after CRT is independently associated with markedly inferior progression-free survival, that ctHPV DNA clearance kinetics predict treatment response, and that molecular detection of residual disease precedes radiographic recurrence by a median of 3–6 months (12, 15, 16). These data have generated intense interest in whether ctHPV DNA-guided risk stratification can enable a paradigm of recurrence-directed intervention: treating molecular relapse before it becomes clinically evident, rather than waiting for radiographic progression.

In this review, we provide a comprehensive synthesis of the biological, technological, and clinical dimensions of MRD in cervical cancer. We first examine the molecular mechanisms by which cervical cancer cells evade definitive chemoradiotherapy and establish persistent residual disease (Section 2). We then evaluate the evidence supporting ctHPV DNA as a liquid biopsy biomarker for post-treatment surveillance, including assay performance characteristics, optimal sampling schedules, and clinical validation data (Section 3). Next, we critically appraise emerging strategies for recurrence-directed intervention, from MRD-stratified intensification of standard therapy to ctHPV DNA-guided deployment of immunotherapy, therapeutic vaccination, and adoptive cell therapy (Section 4). In the Discussion (Section 5), we synthesize cross-cutting themes, address knowledge gaps and trial design considerations, and situate MRD in cervical cancer within the broader liquid biopsy and immunotherapy landscape. We conclude by outlining a unifying framework and the translational roadmap required to realize MRD-guided precision surveillance in cervical cancer.

## 2 Biological basis of minimal residual disease after chemoradiotherapy

### 2.1 HPV integration, viral oncogene addiction, and clonal persistence

HPV integration into the host genome is a pivotal molecular event in cervical carcinogenesis that also shapes the biology of residual disease after treatment. In the normal viral life cycle, HPV exists as an extrachromosomal episome within the nucleus of infected basal epithelial cells, with viral gene expression tightly coupled to host cell differentiation (17). Integration—which occurs in >80% of invasive cervical cancers—disrupts the viral E1/E2 open reading frame, releasing the E6 and E7 oncogenes from E2-mediated transcriptional repression and enabling their constitutive, high-level expression (17, 18). This integration event is stochastic with respect to host genomic location but recurrently targets fragile sites and transcriptionally active regions, resulting in copy-number alterations, structural rearrangements, and focal amplifications at integration loci (19).

The oncogenic consequences of integration extend beyond E6/E7 dysregulation. Integration sites are frequently enriched near cancer-relevant genes, and the process can generate HPV–host fusion transcripts with oncogenic potential (18). Furthermore, integration stabilizes the malignant phenotype: once integrated, the viral oncogenes become permanent residents of the cancer genome, transmitted to daughter cells through mitosis. This genomic fixation of the viral driver has two critical implications for MRD biology. First, residual tumor cells that survive chemoradiotherapy continue to depend on E6/E7 expression for proliferation and survival—a state of “viral oncogene addiction” that renders them persistently vulnerable to HPV-directed therapies (11, 20). Second, the integrated viral DNA serves as a stable, clone-specific molecular barcode: ctHPV DNA detected in plasma after treatment is necessarily derived from tumor cells and not from normal tissues or lytic viral infection, because integrated HPV DNA is not a feature of productive viral replication (16).

The clonal architecture of residual disease after chemoradiotherapy remains incompletely characterized. Chemoradiotherapy imposes a profound selective bottleneck: radiation induces double-strand DNA breaks, while cisplatin generates intra- and interstrand crosslinks, both preferentially affecting proliferating cells (21). However, tumor cell subpopulations with intrinsic or acquired resistance—conferred by hypoxia, enhanced DNA damage repair capacity, cancer stem cell-like properties, or quiescence—can survive this bottleneck and serve as the reservoir for subsequent recurrence (22). In cervical cancer specifically, hypoxia is a well-established adverse prognostic factor that reduces radiosensitivity through multiple mechanisms, including stabilization of HIF-1α, metabolic reprogramming toward glycolysis, and selection of apoptosis-resistant clones (23). The proportion of hypoxic tumor volume on pretreatment imaging has been associated with inferior local control after radiotherapy, suggesting that hypoxic subregions may be a principal niche for MRD persistence (23).

### 2.2 Immune evasion and the post-treatment tumor microenvironment

Persistent high-risk HPV infection creates a complex immunological paradox: the constitutive expression of viral oncoproteins—which are obligate foreign antigens—should render HPV-transformed cells highly susceptible to T-cell-mediated immune clearance, yet cervical cancers routinely evade immune surveillance and progress to invasive disease (24, 25). This paradox is resolved by an array of immune evasion mechanisms that are reinforced and often amplified by chemoradiotherapy.

The HPV E6 and E7 oncoproteins actively suppress innate immune signaling. E6 inhibits the type I interferon (IFN) response by interfering with IRF-3 activation and STAT-1 signaling, while E7 downregulates Toll-like receptor 9 (TLR9) expression and inhibits the cGAS-STING DNA-sensing pathway (24, 26). These viral strategies are complemented by the selection of host-cell adaptations during carcinogenesis, including loss of HLA class I expression (which abrogates CD8+ T-cell recognition of viral peptide–MHC complexes), upregulation of PD-L1 in response to IFN-γ in the tumor microenvironment, and recruitment of immunosuppressive regulatory T cells (Tregs) and myeloid-derived suppressor cells (MDSCs) (25, 27).

Chemoradiotherapy exerts dual and potentially antagonistic effects on anti-tumor immunity. On one hand, radiation-induced immunogenic cell death releases tumor antigens—including E6/E7-derived peptides—and damage-associated molecular patterns (DAMPs) such as calreticulin, HMGB1, and ATP, which can stimulate dendritic cell maturation and cross-priming of tumor-specific CD8+ T cells (28). The abscopal effect, in which localized radiation induces regression of distant, unirradiated lesions, provides clinical evidence for this immunostimulatory dimension. On the other hand, fractionated radiation also depletes circulating lymphocytes, induces lymphopenia-associated homeostatic proliferation of Tregs, and promotes TGF-β-mediated fibrosis and immune exclusion within the irradiated field (29, 30). Persistent residual tumor cells after CRT are thus immersed in a post-treatment microenvironment characterized by relative immune paucity, fibrosis, and the predominance of immunosuppressive over effector immune subsets—conditions that may facilitate the outgrowth of residual clones.

Recent single-cell and spatial transcriptomic analyses of cervical cancer have begun to resolve the cellular dynamics of immune evasion during disease progression. Guo et al. applied integrated single-cell RNA sequencing and spatial transcriptomics to samples spanning normal cervix, HPV-infected precancer, and invasive carcinoma, revealing progressive depletion of tissue-resident CD8+ T cells and NK cells, expansion of exhausted CD8+ T cells expressing PD-1, CTLA-4, and TIM-3, and enrichment of immunosuppressive cancer-associated fibroblasts (CAFs) in the invasive front (14). These data suggest that by the time of clinical presentation, cervical cancers have already established a deeply immunosuppressed microenvironment that may be partially disrupted but not durably reversed by standard CRT. The extent to which the post-CRT immune milieu can be remodeled by immunotherapy to target MRD is a critical open question that underpins current adjuvant checkpoint inhibitor trials.

### 2.3 Tumor dormancy and the kinetics of recurrence

The temporal pattern of cervical cancer recurrence—with a predominant peak at 12–24 months after treatment and a long tail extending beyond 5 years—is consistent with a model in which residual tumor cells enter a state of proliferative quiescence (cellular dormancy) or are held in equilibrium by immune surveillance (immunological dormancy) before eventual outgrowth (31). The molecular mechanisms governing entry into and exit from dormancy in cervical cancer are poorly understood, but insights from other epithelial malignancies suggest contributions from microenvironmental cues (hypoxia, nutrient deprivation, extracellular matrix stiffness), epigenetic reprogramming, and the balance between pro-apoptotic and survival signals downstream of DNA damage (22, 32).

The concept of tumor dormancy has direct therapeutic implications for MRD-directed intervention. If residual cervical cancer cells are quiescent, they may be refractory to cytotoxic agents that target proliferating cells—a consideration that may partially explain the failure of adjuvant chemotherapy in the OUTBACK trial, where four cycles of adjuvant carboplatin–paclitaxel after CRT did not improve overall survival (3). Conversely, immunotherapeutic approaches that do not depend on target-cell proliferation—such as checkpoint blockade, which relieves exhaustion of pre-existing tumor-reactive T cells, or adoptive transfer of ex vivo-expanded tumor-infiltrating lymphocytes—may be better suited to eliminate dormant residual disease (19, 33).

Understanding the transition from MRD to clinical recurrence requires longitudinal profiling of residual disease through its molecular evolution. ctHPV DNA kinetics provide a non-invasive window into this process: pretreatment ctHPV DNA levels reflect total-body tumor burden, post-treatment clearance rates reflect the rapidity of tumor cell kill, and the emergence of detectable ctHPV DNA after an initial negative period—so-called molecular recurrence—presumably reflects the exit of a previously dormant clone into proliferative expansion (12, 15). The lead time between molecular and clinical recurrence observed across multiple studies (~3–6 months for cervical cancer, ~3.9 months for HPV+ oropharyngeal cancer) suggests a window of opportunity for intervention at a stage of lower disease burden, when therapeutic efficacy may be higher and when tumor heterogeneity and treatment resistance may be less entrenched (12, 13, 16).

## 3 ctHPV DNA as a liquid biopsy biomarker for post-treatment surveillance

### 3.1 Analytical platforms: digital PCR and next-generation sequencing

The detection and quantification of ctHPV DNA in plasma presents distinct analytical challenges and opportunities relative to mutation-based ctDNA assays. The HPV genome is small (~8 kb double-stranded circular DNA) and encodes well-characterized, genotype-specific sequences within the E6 and E7 open reading frames that can be targeted by PCR primers or capture probes with high specificity (10). Because the same viral genotype is present in all tumor cells of a given patient (in contrast to patient-specific somatic mutations that must first be identified through tumor sequencing), HPV-targeted assays can be deployed in a tumor-agnostic manner—without requiring prior knowledge of the patient’s tumor mutational profile (12, 16).

Two principal analytical strategies have been validated for ctHPV DNA detection. Droplet digital PCR (ddPCR) partitions individual DNA molecules into nanoliter-scale droplets, each containing a fluorescent probe-based PCR reaction; endpoint fluorescence is read for each droplet, enabling absolute quantification of target molecules without reliance on standard curves. ddPCR assays targeting HPV16, HPV18, and other high-risk genotypes have demonstrated limits of detection (LoD) in the range of 0.1–0.5 copies per milliliter of plasma and high analytical specificity, with negligible cross-reactivity across genotypes (15, 34). Fernholz et al. recently described a comprehensive ddPCR panel covering >14 high-risk HPV genotypes, demonstrating that genotype-specific quantification in plasma accurately reflects tumor HPV status confirmed by tissue-based testing (35). Matsunaga et al. applied type-specific ddPCR to preoperative plasma from surgically resected cervical cancer patients, showing that ctHPV DNA detectability correlated with tumor size, lymphovascular space invasion, and nodal metastasis (34).

Next-generation sequencing (NGS)-based approaches offer complementary advantages, including the ability to detect HPV genotypes without *a priori* knowledge of which genotype to target and the potential for higher sensitivity through the aggregate interrogation of multiple genomic regions (16, 36). Bønløkke et al. developed and validated an NGS-based assay that enriches for HPV-derived cell-free DNA fragments using HPV-specific capture probes, then sequences the captured library to identify and quantify HPV reads (16). In a prospective study of 141 cervical cancer patients, this HPV-agnostic approach achieved a LoD of approximately 0.02–0.05 copies/mL and detected ctHPV DNA in 87% of pretreatment samples, with sensitivity varying by stage (67% for stage I, 92% for stage II–IV) (16). Han et al. used a similar HPV-seq approach alongside ddPCR in a prospective validation cohort of 70 patients, demonstrating strong concordance between the two methods and confirming that both independently predicted progression-free survival (12).

The choice between ddPCR and NGS platforms involves trade-offs in sensitivity, breadth of genotype coverage, cost, and workflow complexity. ddPCR is simpler, faster, and less expensive per sample, making it well-suited for high-volume surveillance applications where the tumor HPV genotype is known. NGS-based approaches offer broader genotype coverage (important for the ~10% of cervical cancers driven by less common HPV types) and capture-level multiplexing that enables simultaneous analysis of HPV and host genomic alterations (36). The two methodologies are not mutually exclusive and may be deployed in complementary fashion: ddPCR for routine surveillance, with NGS reserved for confirmatory testing or for cases where the tumor HPV genotype is unknown.

### 3.2 Pre-analytical variables and optimal sampling schedules

The translation of ctHPV DNA testing from research cohorts to clinical practice requires standardization of pre-analytical variables that influence assay performance. Cell-free DNA is intrinsically fragile, with a half-life in circulation of approximately 16 minutes to 2.5 hours (37). Delays between venipuncture and plasma separation permit the release of genomic DNA from lysed leukocytes, diluting the tumor-derived fraction and potentially causing false-negative results. Standardized protocols using cell-stabilizing blood collection tubes (e.g., Streck or PAXgene tubes) that permit sample processing within 48–72 hours have been adopted across most prospective studies (12, 16).

The timing of post-treatment ctHPV DNA assessment critically influences its prognostic performance. Based on available data from both cervical cancer and the more extensively studied HPV+ oropharyngeal cancer setting, a consensus is emerging around several key timepoints. End-of-treatment (EOT) ctHPV DNA (measured within 1–2 weeks of CRT completion) reflects the immediate tumor cell kill achieved by therapy and identifies patients with gross residual disease; Han et al. reported that detectable EOT ctHPV DNA was associated with a 2-year PFS of 51% versus 77% for those with undetectable levels (12). Early post-treatment ctHPV DNA (4–6 weeks after CRT) provides a more clinically actionable window: patients with detectable ctHPV DNA at this timepoint had a 2-year PFS of 15% versus 82% for those with undetectable levels in the Han et al. study, and Bønløkke et al. reported an adjusted HR of 19.1 for recurrence in patients with positive ctHPV DNA at the first post-treatment sample (12, 16). Serial surveillance (every 3 months for the first 2 years) enables detection of molecular recurrence before clinical relapse; in the Bønløkke study, ctHPV DNA became detectable before clinical evidence of recurrence in 61% of relapsing patients, with a mean lead time of 144 days (16).

These data support a sampling framework in which baseline (pretreatment), EOT, 4–6 weeks post-CRT, and then serial 3-monthly ctHPV DNA assessments constitute a rational surveillance schedule. Harmonization of this schedule across ongoing and future trials will be essential for comparability and for establishing evidence-based guidelines.

### 3.3 Clinical validation of ctHPV DNA as a prognostic biomarker

The clinical utility of ctHPV DNA for post-treatment surveillance in cervical cancer has been established through a series of progressively larger and more rigorous prospective studies over the past five years. The evidence base is now anchored by two landmark studies.

Han et al. (2024) reported a prospective, multicenter validation study in 70 patients with HPV+ stage IB–IVA cervical cancer treated with definitive CRT (12). Plasma was collected at baseline, EOT, 4–6 weeks, and 3 months post-CRT and analyzed by both ddPCR and HPV-seq. The key findings were as follows: (i) ctHPV DNA clearance by EOT was observed in 84% of patients; (ii) detectable ctHPV DNA at 4–6 weeks post-CRT was the strongest predictor of recurrence (2-year PFS: 15% vs 82%, p < 0.001); (iii) on multivariable analysis adjusting for stage, nodal status, and histology, post-treatment ctHPV DNA remained independently associated with inferior PFS; (iv) the HPV-seq assay (LoD 0.023 copies/mL) detected ctHPV DNA in 3 patients that were negative by ddPCR (LoD 0.18 copies/mL), all of whom subsequently recurred, suggesting that ultrasensitive detection captures additional high-risk patients; and (v) the median lead time from ctHPV DNA detection to radiographic recurrence was 5.9 months (12). This study represents the most rigorous clinical validation of ctHPV DNA for MRD detection in cervical cancer to date and provides the strongest evidence for its incorporation into post-treatment surveillance.

Bønløkke et al. (2026) reported an NGS-based approach in 141 cervical cancer patients encompassing all stages (IA1–IVB), treated with surgery, radiotherapy, or CRT (16). Key findings included: (i) pretreatment ctHPV DNA positivity rate of 87% overall; (ii) ctHPV DNA positivity at the first post-treatment sample (FPS, collected a median of 3.4 months after treatment) was associated with an adjusted HR of 19.1 (95% CI 7.27–50.2) for recurrence; (iii) positive predictive value (PPV) of 83% and negative predictive value (NPV) of 90% for recurrence within 50 months; (iv) ctHPV DNA dynamics from pre- to post-treatment (the ratio of post- to pretreatment levels) added prognostic information beyond static post-treatment measurements; and (v) the assay detected ctHPV DNA without requiring prior knowledge of the tumor HPV genotype, making it broadly applicable across the HPV genotype spectrum (16). This study extended the evidence base beyond the CRT-only population to include surgically managed patients and demonstrated the feasibility of HPV-agnostic surveillance.

Additional studies have contributed important corroborating evidence. Mittelstadt et al. developed a ddPCR panel covering 13 HPV types and demonstrated that serial ctHPV DNA measurements tracked treatment response; in their cohort, ctHPV DNA levels decreased by >99% in patients achieving complete remission, while persistent or rising levels preceded clinical relapse (15). Thangarajah et al. showed that ddPCR-based ccfHPV-DNA quantification in cervical and vulvar cancer patients correlated with clinical course, with levels dropping below detection in patients achieving remission and rising at recurrence (38). A meta-analysis by Karimi et al. encompassing 16 studies of HPV-associated cancers (cervical and oropharyngeal) reported a pooled HR of 7.97 (95% CI 3.56–17.87) for recurrence in patients with detectable post-treatment ctHPV DNA, confirming the consistency of this association across study populations and tumor types (39). Broader evidence further supports circulating cell-free HPV DNA as a diagnostic and prognostic biomarker in cervical cancer, including meta-analytic evidence, assay-specific studies, systematic reviews, and recent clinical investigations across precancerous, primary, and recurrent or metastatic disease settings (8, 40–46).

It is instructive to compare the cervical cancer ctHPV DNA literature with the more mature evidence base in HPV+ oropharyngeal squamous cell carcinoma (OPSCC). The landmark study by Chera et al. (2020) in 115 patients with HPV+ OPSCC demonstrated that ctHPV DNA surveillance using a ddPCR assay achieved an NPV of 100%—87 patients with persistently undetectable ctHPV DNA after treatment experienced zero recurrences—and a PPV of 94% for two consecutively positive tests, with a median lead time of 3.9 months (13). Subsequent studies in OPSCC have reinforced these findings and have begun to explore ctHPV DNA-guided treatment de-escalation strategies (47). The OPSCC experience provides both a proof of principle for ctHPV DNA-guided surveillance and a cautionary note: the NPV in cervical cancer (90%) has not yet matched the near-perfect NPV observed in OPSCC, likely reflecting differences in tumor biology, treatment regimens, and assay performance across anatomical sites.

### 3.4 Integration with conventional surveillance modalities

The relationship between ctHPV DNA testing and conventional post-treatment surveillance modalities—physical examination, cervical cytology, and imaging—warrants careful consideration. These modalities are not mutually exclusive but provide complementary information, and ctHPV DNA should be evaluated not as a replacement for existing methods but as an adjunct that addresses their principal limitation: the inability to detect subclinical residual disease.

FDG-PET/CT performed at 3 months after CRT has a sensitivity of approximately 70–85% and a specificity of 75–90% for detecting residual or recurrent disease, but its NPV is imperfect, and false-positive results due to post-radiation inflammation lead to unnecessary investigations (6). The HPV-seq study by Han et al. included a subset analysis comparing ctHPV DNA with 3-month post-treatment FDG-PET/CT; ctHPV DNA positivity identified all patients who subsequently recurred and correctly classified 3 patients with indeterminate PET findings as true negatives who did not recur (12). These data, while preliminary, suggest that ctHPV DNA testing may resolve equivocal imaging findings and enhance the specificity of post-treatment surveillance.

Cervical cytology and high-risk HPV DNA testing from cervical samples are well established for post-treatment surveillance of cervical intraepithelial neoplasia (CIN). However, their role after definitive CRT for invasive cervical cancer is limited: radiation-induced cytological atypia can complicate interpretation, while cervical sampling may fail to detect recurrences arising in the pelvic sidewall, regional lymph nodes, or distant organs because such lesions may not involve the cervical canal and therefore may not shed detectable tumor-derived material into cervical samples (48). ctHPV DNA testing may complement local cervical sampling by detecting tumor-derived HPV DNA released into the systemic circulation, including from disease sites beyond the cervix.

A practical algorithm integrating ctHPV DNA into post-treatment surveillance would involve: (i) baseline ctHPV DNA quantification at diagnosis; (ii) EOT and early post-treatment (4–6 week) assessments to identify patients with molecular evidence of residual disease who warrant intensified surveillance or early intervention; (iii) serial 3-monthly assessments for the first 2 years to detect molecular recurrence; and (iv) triggered imaging (FDG-PET/CT) for patients with rising or persistently positive ctHPV DNA to localize disease and guide intervention. This approach, while rational, requires validation in prospective interventional trials that randomize patients to ctHPV DNA-guided versus standard surveillance.

## 4 Recurrence-directed intervention: from risk stratification to MRD-guided treatment

### 4.1 The rationale for MRD-directed treatment intensification

The distinction between risk-stratified adjuvant therapy (administered to all patients meeting clinical risk criteria, regardless of MRD status) and MRD-directed therapy (administered only to patients with molecular evidence of residual disease) is central to the evolving paradigm of personalized post-treatment management. Risk-stratified approaches, exemplified by the OUTBACK and INTERLACE trials, apply treatment intensification to broad populations based on pretreatment risk features. The OUTBACK trial demonstrated that unselected adjuvant carboplatin–paclitaxel after CRT did not improve overall survival (5-year OS: 72% vs 71%, HR 0.90, p = 0.81) and increased toxicity, effectively closing the door on empiric adjuvant chemotherapy in this setting (3). The INTERLACE trial, by contrast, demonstrated a statistically significant overall survival benefit for induction chemotherapy administered before CRT (5-year OS: 80% vs 72%, HR 0.61, p = 0.04) (49). The divergence of these two trials—both testing chemotherapy intensification albeit at different points in the treatment sequence—illustrates the importance of timing and the potential futility of intensifying therapy after CRT in patients who have already been cured.

MRD-directed approaches offer a fundamentally different strategy: treat only the minority of patients who have molecular evidence of persistent disease, sparing the cured majority from unnecessary toxicity. This paradigm has been most extensively validated in colorectal cancer, where ctDNA-guided adjuvant chemotherapy after surgery has been shown to reduce chemotherapy use without compromising outcomes in patients with undetectable postoperative ctDNA, while identifying a high-risk subgroup most likely to benefit from treatment intensification (50, 51). The extension of this paradigm to cervical cancer is conceptually appealing but requires evidence that (i) ctHPV DNA positivity after CRT reliably identifies patients destined to recur, (ii) interventions initiated at the time of molecular recurrence improve outcomes compared with treatment at the time of clinical recurrence, and (iii) the lead time afforded by ctHPV DNA surveillance is sufficient to permit effective intervention.

### 4.2 Immune checkpoint inhibition in the post-CRT setting

The integration of immune checkpoint inhibitors into the definitive treatment of locally advanced cervical cancer represents the most significant therapeutic advance in this disease since the establishment of concurrent cisplatin-based CRT in 1999. The KEYNOTE-A18 trial demonstrated that adding pembrolizumab to CRT and continuing it as maintenance therapy for 15 cycles significantly improved both progression-free survival and overall survival in patients with high-risk locally advanced disease (52). At a median follow-up of 29.9 months, the 36-month OS was 82.6% with pembrolizumab versus 74.8% with placebo (HR 0.67, p = 0.0040), establishing pembrolizumab-based immuno-chemoradiotherapy as a new standard of care (52).

For the purposes of MRD-directed therapy, KEYNOTE-A18 demonstrates proof of principle that post-CRT immunotherapy can impact distant failure rates and survival. However, it is important to note that KEYNOTE-A18 tested a risk-stratified rather than an MRD-directed strategy: pembrolizumab was administered to all patients meeting high-risk clinical criteria, not selectively to those with molecular evidence of residual disease. The CALLA trial, which tested durvalumab (anti-PD-L1) concurrent with and following CRT, did not meet its primary endpoint of improving PFS, underscoring that not all checkpoint inhibitors have equivalent activity in this context and that patient selection matters (53).

The most compelling opportunity for MRD-directed checkpoint inhibition lies in the window between ctHPV DNA detection and clinical recurrence. If patients with detectable post-CRT ctHPV DNA have a 2-year PFS of 15–24% (as demonstrated by Han et al. (12)), then this molecularly defined population has an urgent need for effective intervention. Pembrolizumab—already established as active in both the first-line (KEYNOTE-826) and second-line (KEYNOTE-158) settings—is a logical candidate for evaluation in this context (54, 55). A clinical trial randomizing patients with detectable ctHPV DNA at 4–6 weeks post-CRT to pembrolizumab maintenance versus observation, with ctHPV DNA clearance as an intermediate endpoint, would directly test the MRD-directed treatment hypothesis. The broader systemic-treatment landscape in recurrent or metastatic cervical cancer has also demonstrated survival benefits with bevacizumab-containing therapy and, in previously treated disease, the PD-1 inhibitor cemiplimab (56, 57).

### 4.3 Therapeutic HPV vaccination: targeting viral oncogene persistence

Therapeutic vaccination against HPV E6 and E7 oncoproteins represents a mechanistically distinct strategy for targeting MRD that is uniquely enabled by the viral etiology of cervical cancer. Unlike prophylactic HPV vaccines (which target the L1 capsid protein to prevent infection), therapeutic vaccines aim to generate or amplify T-cell responses against E6/E7-derived peptides presented on MHC class I molecules on the surface of HPV-transformed cells (20, 58).

The clinical proof of principle for therapeutic HPV vaccination was established by Kenter et al. in a landmark phase II trial of a synthetic long-peptide vaccine (SLP) encompassing HPV16 E6 and E7 epitopes in patients with HPV16+ vulvar intraepithelial neoplasia (VIN) grade 3 (59). At 12 months, 79% of vaccinated patients experienced clinical responses, including 47% with complete regression, and clinical benefit was strongly correlated with the magnitude of vaccine-induced HPV-specific CD4+ and CD8+ T-cell responses (59). Subsequent trials of SLP vaccines in cervical cancer have shown immunological activity, with the generation of robust HPV-specific T-cell responses, but clinical efficacy in the invasive cancer setting has been more modest (60, 61).

Two considerations make therapeutic vaccination particularly promising as an MRD-directed strategy. First, the target antigens (E6 and E7) are obligate drivers of the malignant phenotype—they cannot be downregulated or lost by tumor cells without compromising fitness—and are expressed uniformly in HPV-driven cancers (11, 20). Second, vaccination is likely to be most effective in the low-disease-burden setting of MRD, where the ratio of vaccine-induced effector T cells to target tumor cells is favorable, and where the immunosuppressive tumor microenvironment—which can blunt vaccine efficacy in the setting of bulky disease—has been partially disrupted by prior CRT. A clinical trial evaluating therapeutic HPV vaccination in patients with detectable post-CRT ctHPV DNA, with ctHPV DNA clearance as the primary endpoint, would directly test this hypothesis.

The translational pipeline for therapeutic HPV vaccines has expanded substantially, encompassing DNA vaccines (VGX-3100), viral vector-based vaccines (ADXS11-001/axalimogene filolisbac, a live attenuated Listeria monocytogenes vector encoding HPV16 E7 fused to listeriolysin O), RNA-based vaccines, and dendritic cell-based approaches (20, 58). The mRNA-based vaccine platform, whose clinical feasibility and scalability have been demonstrated by the COVID-19 pandemic, is particularly well suited to MRD-directed vaccination: mRNA vaccines encoding E6 and E7 elicit potent CD8+ T-cell responses in preclinical models and can be rapidly manufactured on a patient-specific basis (62). Combining therapeutic vaccination with checkpoint blockade—to overcome PD-1/PD-L1-mediated T-cell exhaustion that may limit vaccine efficacy—is an active area of investigation that is directly relevant to the post-CRT MRD setting (63).

### 4.4 Adoptive cell therapy and the frontier of personalized MRD treatment

Adoptive cell therapy (ACT) using tumor-infiltrating lymphocytes (TILs) has produced some of the most dramatic therapeutic outcomes in cervical cancer, including durable complete responses in patients with chemotherapy-refractory metastatic disease. Stevanovic et al. at the National Cancer Institute reported that infusion of autologous TILs selected for HPV E6/E7 reactivity—following a lymphodepleting preparative regimen and followed by systemic high-dose interleukin-2—induced objective responses in 28% of heavily pretreated cervical cancer patients, with two complete responses ongoing beyond 5 years (19, 64). These results established that adoptively transferred T cells can mediate the regression of an epithelial malignancy and that HPV viral proteins are therapeutically actionable tumor rejection antigens.

The application of TIL therapy to the MRD setting faces substantial practical barriers—including the requirement for tumor tissue for TIL expansion (which is problematic after CRT), the complexity and cost of manufacturing, and the toxicity of the high-dose IL-2 regimen—that currently limit its deployment to specialized centers and to patients with measurable disease. However, T-cell receptor-engineered T cells (TCR-T) targeting defined HPV16 E6 or E7 epitopes presented by common HLA alleles (e.g., HLA-A*02:01) are under active clinical development and may offer a more readily deployable cell therapy product (65). TCR-T cells can be manufactured from peripheral blood without requiring tumor tissue, and when directed against shared viral antigens rather than patient-specific neoantigens, they can be generated as off-the-shelf products for defined HLA–antigen combinations.

In the MRD context, the most compelling application of ACT may be as consolidation therapy for patients with molecular evidence of persistent disease after CRT who have failed or are ineligible for less intensive interventions (checkpoint inhibition, therapeutic vaccination). The extraordinary potency of ACT—capable of mediating complete tumor regression even in bulky, chemorefractory disease—may be best deployed against the lowest disease burden, where the probability of cure is highest and the toxicity–benefit calculus most favorable.

### 4.5 Antibody–drug conjugates and chemotherapy in the MRD context

The antibody–drug conjugate (ADC) tisotumab vedotin, which targets tissue factor (TF) expressed on cervical cancer cells and delivers the microtubule-disrupting payload monomethyl auristatin E (MMAE), received accelerated FDA approval based on the innovaTV 204 phase II trial, which demonstrated an objective response rate of 24% in previously treated recurrent/metastatic cervical cancer (66). The subsequent confirmatory phase III innovaTV 301 trial met its primary endpoint of improved overall survival, leading to full regulatory approval (67). While tisotumab vedotin is currently positioned in the late-line recurrent/metastatic setting, its activity in pretreated disease and its distinct mechanism of action—which does not depend on immune competence or HPV-directed immune recognition—make it a candidate for evaluation in the MRD setting should immunotherapy-based approaches prove insufficient.

Conventional chemotherapy in the MRD context faces the challenge that residual tumor cells may be quiescent and thus resistant to agents that target proliferating cells—a consideration that may partially explain the neutral results of the OUTBACK trial (3). However, chemotherapy combined with immunotherapy (as in KEYNOTE-826) or deployed as induction rather than adjuvant treatment (as in INTERLACE) has demonstrated clinical benefit, suggesting that the specific treatment sequence, patient selection, and combinatorial strategy—rather than chemotherapy per se—determine efficacy in the post-CRT setting (49, 54).

### 4.6 A unifying framework: ctHPV DNA-guided, immune-based MRD interception

Synthesizing the evidence reviewed above, we propose a conceptual framework for MRD-directed management in cervical cancer in which ctHPV DNA dynamics serve as the biomarker foundation, and where the treatment strategy is tailored to individual risk, tumor HPV genotype, and immunological context. The framework proceeds through sequential decision nodes: (1) Pretreatment risk stratification: all patients undergo baseline ctHPV DNA quantification and HPV genotyping. Patients are stratified by clinical risk (stage, nodal status, histology) and by ctHPV DNA level, which reflects total-body tumor burden (12, 16). (2) Definitive treatment: patients with locally advanced disease receive curative-intent CRT, which may be preceded by induction chemotherapy (per INTERLACE) or combined with pembrolizumab (per KEYNOTE-A18) (49, 52). (3) Early post-treatment MRD assessment: ctHPV DNA is measured at EOT and at 4–6 weeks post-CRT. Patients with undetectable ctHPV DNA (approximately 80–85% of the population) are classified as molecular complete responders and enter standard surveillance (12). Patients with detectable ctHPV DNA (15–20%) are classified as molecular residual disease-positive and are candidates for MRD-directed intervention. (4) MRD-directed intervention: for molecular residual disease-positive patients, options include immune checkpoint inhibition (pembrolizumab maintenance), therapeutic HPV vaccination targeting the patient’s tumor HPV genotype, or for highest-risk patients (persistently positive ctHPV DNA despite checkpoint inhibition), adoptive cell therapy on a clinical trial. (5) Longitudinal molecular surveillance: all patients undergo serial ctHPV DNA monitoring every 3 months for 2 years; molecular recurrence triggers FDG-PET/CT for localization and salvage therapy. ctHPV DNA clearance serves as an intermediate endpoint to assess the efficacy of MRD-directed interventions.

This framework is aspirational rather than immediately actionable: multiple components require prospective validation, and the optimal MRD-directed intervention has not been defined. However, each component is supported by at least one level of clinical evidence, and the framework is designed to be testable in modular fashion through clinical trials.

## 5 Discussion

The evidence synthesized in this review supports the central thesis that MRD in cervical cancer is a biologically tractable and clinically consequential phenomenon whose detection and therapeutic targeting are now within reach. However, several cross-cutting themes emerge from our analysis that merit extended discussion: the relationship between assay sensitivity and clinical utility, the interpretation of ctHPV DNA dynamics across heterogeneous treatment modalities, the biological determinants of treatment responsiveness in the MRD setting, and the translational challenges that must be overcome to realize MRD-guided management in routine clinical practice.

### 5.1 Sensitivity–specificity trade-offs and the clinical significance of ultra-low ctHPV DNA

One of the most important unresolved questions in the field concerns the clinical significance of ctHPV DNA detected at concentrations below the LoD of ddPCR (~0.1–0.5 copies/mL) but within the reach of ultrasensitive NGS-based assays (~0.02 copies/mL). In the Han et al. study, three patients with ctHPV DNA detectable exclusively by HPV-seq subsequently recurred, suggesting that ultra-sensitive detection captures clinically relevant residual disease (12). However, the broader natural history of ultra-low-level ctHPV DNA—whether it invariably progresses to clinical recurrence, can be spontaneously cleared by host immunity, or can enter long-term dormancy—remains unknown. This question has direct clinical implications: if a substantial proportion of patients with ultra-low ctHPV DNA would never develop clinical recurrence, then deploying assays with maximal sensitivity will generate false-positive molecular results that provoke unnecessary intervention and anxiety, eroding the clinical utility of MRD testing.

The colorectal cancer ctDNA literature offers instructive parallels. In the DYNAMIC trial, patients with stage II colon cancer and detectable postoperative ctDNA who received adjuvant chemotherapy had superior outcomes compared with those who did not, establishing the clinical validity of ctDNA-guided treatment decision-making (51). However, the optimal ctDNA threshold for treatment initiation, and whether patients with extremely low ctDNA levels benefit from the same degree of treatment intensification as those with higher levels, remain open questions. In cervical cancer, defining the ctHPV DNA threshold that optimally balances sensitivity (detecting all patients destined to recur) against specificity (avoiding over-treatment of patients whose residual disease would be spontaneously controlled) is a high-priority research objective that will require prospective observational cohorts with extended follow-up and careful annotation of molecular and clinical outcomes.

### 5.2 Cross-cancer comparisons: lessons from HPV+ oropharyngeal cancer and colorectal cancer

The parallel development of ctHPV DNA surveillance in HPV+ OPSCC provides both validation of the general approach and important lessons regarding anatomical site-specific differences. The near-perfect NPV of 100% reported by Chera et al. in OPSCC has not been replicated in cervical cancer, where NPV ranges from 82% to 90% across studies (12, 13, 16). Several factors may account for this discrepancy. First, the anatomical site of residual disease influences ctDNA shedding: pelvic recurrences in cervical cancer, especially those confined to the previously irradiated field, may release less tumor DNA into the systemic circulation than the more vascularized oropharyngeal and cervical nodal recurrences typical of OPSCC. Second, the tumor volume threshold for ctDNA detection may differ between these diseases due to differences in tumor vascularity, cell turnover rate, and the proportion of tumor DNA released as cell-free fragments. Third, the lower NPV in cervical cancer may reflect a higher rate of HPV-independent or HPV-low recurrences in which viral DNA is not an adequate surrogate for tumor burden; such cases, while rare, would manifest as false-negative ctHPV DNA results.

The colorectal cancer ctDNA experience highlights a further consideration: the clinical utility of MRD testing is ultimately determined not by its prognostic performance alone, but by whether MRD-directed interventions improve outcomes. In colorectal cancer, the demonstration that ctDNA-guided adjuvant chemotherapy reduces treatment without compromising survival—and that ctDNA-positive patients benefit from intensification—has been established through prospective randomized trials (50, 51). The cervical cancer field has not yet produced analogous interventional data, and the design and execution of such trials should be the highest priority for the next phase of research in this area. The INTERLACE and KEYNOTE-A18 trials, while not MRD-directed, provide proof of concept that treatment intensification anchored to pretreatment risk features can improve survival in locally advanced cervical cancer, lending plausibility to the hypothesis that post-treatment MRD-based intensification may confer similar or greater benefit with improved patient selection (49, 52).

### 5.3 The immunological context of MRD: implications for treatment selection

The immunological state of residual disease after CRT—whether residual tumor cells are under active immune surveillance, have induced T-cell exhaustion, or reside in an immunologically privileged niche—is likely to be a critical determinant of which MRD-directed intervention is most appropriate for a given patient. Checkpoint inhibitors (anti-PD-1/PD-L1) are most effective when pre-existing tumor-reactive T cells are present but functionally impaired by PD-1/PD-L1 signaling; therapeutic HPV vaccines are most effective when HPV-specific T-cell precursors exist but have not been sufficiently expanded; and adoptive cell therapy bypasses the requirement for endogenous immunity altogether by providing a bolus of ex vivo-expanded effector cells.

Currently, no clinically validated biomarker exists to guide the choice among these immunological modalities in the MRD setting. The integration of ctHPV DNA monitoring with peripheral blood immune profiling—quantification of HPV-specific T-cell frequency and polyfunctionality, circulating cytokine and chemokine profiles, and T-cell receptor repertoire diversity—could enable a more refined stratification of MRD-positive patients and inform the selection of immune-based MRD-directed therapy (68). For example, a patient with detectable post-CRT ctHPV DNA and evidence of HPV-specific T-cell exhaustion (PD-1+Tim-3+ CD8+ T cells) might be optimally treated with pembrolizumab, whereas a patient with detectable ctHPV DNA but absent HPV-specific T-cell responses might benefit most from therapeutic vaccination to prime *de novo* immunity.

### 5.4 Knowledge gaps and trial design considerations

Several fundamental aspects of MRD biology in cervical cancer remain poorly understood and represent high-priority areas for investigation. The clonal composition of residual disease after CRT—whether it reflects the persistence of a dominant treatment-resistant subclone, polyclonal survival across the tumor, or the outgrowth of a minor subclone present at diagnosis—has implications for the design of MRD-directed therapies: a single resistant clone may be targeted with narrow-spectrum approaches, whereas polyclonal residual disease may require broader therapeutic strategies. The application of ultrasensitive ctHPV DNA sequencing, potentially coupled with HPV integration site analysis as a clone-specific identifier, could resolve this question (69).

The immunological correlates of ctHPV DNA clearance versus persistence are largely unexplored. Does spontaneous ctHPV DNA clearance after CRT reflect immunological elimination of residual disease, and if so, what are the effector mechanisms? Do patients with persistent ctHPV DNA harbor defects in HPV-specific T-cell immunity that could be corrected by vaccination or checkpoint blockade? These questions can be addressed by integrating ctHPV DNA monitoring with peripheral blood immune profiling in prospective cohorts (68).

The definitive evaluation of MRD-directed treatment strategies requires innovative clinical trial designs. We outline three designs of escalating ambition: Design 1 (MRD-stratified adjuvant intensification) randomizes patients with detectable ctHPV DNA at 4–6 weeks post-CRT to an MRD-directed intervention versus observation, with ctHPV DNA clearance at 6 months as the primary endpoint. This design requires approximately 100–150 MRD-positive patients (screening population ~600–750). Design 2 (ctHPV DNA-guided versus standard surveillance) randomizes all patients to ctHPV DNA-guided surveillance with triggered imaging and intervention for molecular recurrence versus standard surveillance, with the rate of salvage therapy with curative intent as the primary endpoint. Design 3 (multi-arm MRD platform trial) employs a master protocol that randomizes patients with post-CRT ctHPV DNA positivity to multiple experimental arms (pembrolizumab, therapeutic HPV vaccine, pembrolizumab + vaccine) against a control arm, with ctHPV DNA clearance as an early go/no-go signal and progression-free survival as the definitive endpoint.

### 5.5 Standardization, regulatory pathways, and global health equity

The clinical adoption of ctHPV DNA testing requires standardization across multiple domains. Analytical validation studies should establish the sensitivity, specificity, precision, and limits of detection for each assay platform (ddPCR and NGS) across the full range of clinically relevant HPV genotypes. Inter-laboratory reproducibility studies should ensure that ctHPV DNA results obtained at different centers are comparable. Pre-analytical standards for blood collection, plasma processing, and DNA extraction should be harmonized across clinical laboratories, ideally through a consensus guideline analogous to those developed for mutation-based ctDNA testing in lung and colorectal cancer (70). Regulatory pathways for ctHPV DNA as a biomarker are evolving and will require coordinated effort by academic consortia, commercial assay developers, and regulatory agencies.

Cervical cancer is a disease of global inequity: >85% of cases and ~90% of deaths occur in low- and middle-income countries, where access to radiotherapy, brachytherapy, cisplatin, and immunotherapy is often limited (1, 2). The development of ctHPV DNA-guided management strategies must take this reality into account. ddPCR-based ctHPV DNA testing, which requires relatively modest laboratory infrastructure, may be more readily deployable in resource-limited settings than NGS-based approaches. Point-of-care ctHPV DNA detection platforms would be transformative for global access but remain to be developed. Similarly, the development of affordable MRD-directed strategies—off-patent small-molecule immunomodulators, therapeutic vaccines based on widely accessible nucleic acid platforms, or repurposed generic drugs—should be prioritized alongside the evaluation of premium-priced biologics to ensure that advances in MRD management do not widen existing disparities in cervical cancer outcomes.

### 5.6 Limitations of this review

Several limitations of this review should be acknowledged. First, this is a narrative rather than a systematic review, and the selection and interpretation of evidence reflects the authors’ perspectives. A formal systematic review and meta-analysis would strengthen the evidence base for several of the claims advanced here, particularly regarding the pooled performance characteristics of ctHPV DNA assays and the comparative efficacy of MRD-directed interventions. Second, the literature on ctHPV DNA in cervical cancer is rapidly evolving, with major studies published within the past 18–24 months; several of the landmark trials discussed (KEYNOTE-A18, INTERLACE) have reported only their primary analyses, and longer-term follow-up data may refine the estimates of treatment effect. Third, the framework for MRD-directed management proposed in Section 4.6 represents the authors’ synthesis and has not been validated in prospective clinical studies. Fourth, we have focused principally on ctHPV DNA as the MRD analyte, giving less attention to complementary liquid biopsy approaches—including circulating tumor cells, HPV oncoprotein detection, DNA methylation markers, and extracellular vesicle-based assays—that may provide additional or orthogonal information. Fifth, the scope of this review is limited to invasive cervical cancer; the role of ctHPV DNA in the management of cervical precancer (CIN) and in HPV-associated malignancies at other anatomical sites, while important, is beyond the scope of our analysis.

## 6 Conclusion

Minimal residual disease in cervical cancer is a biologically coherent, technically detectable, and potentially actionable concept that stands at the threshold of clinical translation. The convergence of three advances—the understanding of HPV-driven oncogenesis as a form of viral oncogene addiction that persists in residual tumor cells, the development of ultrasensitive ctHPV DNA detection platforms that can identify molecular residual disease months before radiographic recurrence, and the emergence of immunotherapies capable of amplifying or restoring HPV-directed T-cell immunity—creates the conditions for a paradigm shift in post-treatment management. The evidence reviewed here, anchored by the prospective validation studies of Han et al. (2024) and Bønløkke et al. (2026), supports the contention that ctHPV DNA testing after chemoradiotherapy identifies a high-risk population with residual disease that is invisible to conventional surveillance and that may be amenable to early therapeutic intervention (12, 16).

Nevertheless, substantial work remains before MRD-guided management can be implemented in routine clinical practice. The biological heterogeneity of MRD—its clonal composition, immunological context, and dormancy state—is poorly characterized and likely influences therapeutic responsiveness. The optimal MRD-directed intervention has not been defined and will probably not be singular: checkpoint inhibition, therapeutic vaccination, and adoptive cell therapy address distinct aspects of the tumor–immune interface and may be most effective in combination or in sequence. Prospective interventional trials that randomize patients based on ctHPV DNA status—rather than on pretreatment clinical risk features alone—are urgently needed to move this field from prognostic validation to therapeutic application. The clinical trial designs outlined in this review provide a roadmap for generating this evidence.

Cervical cancer, by virtue of its universal association with a viral driver, is among the most molecularly tractable of solid tumors. The translation of this molecular tractability into improved outcomes for the hundreds of thousands of women diagnosed annually—particularly those in low-resource settings where the burden is highest—is both a scientific imperative and an issue of global health equity. The framework proposed here, in which ctHPV DNA dynamics guide the deployment of immune-based MRD interception, offers a testable path toward this goal.
